# Developing a theory-driven contextually relevant mHealth intervention

**DOI:** 10.1080/16549716.2018.1550736

**Published:** 2019-01-11

**Authors:** Hannah Maria Jennings, Joanna Morrison, Kohenour Akter, Abdul Kuddus, Naveed Ahmed, Sanjit Kumer Shaha, Tasmin Nahar, Hassan Haghparast-Bidgoli, AK Azad Khan, Kishwar Azad, Edward Fottrell

**Affiliations:** a Institute for Global Health, University College London, London, UK; b Perinatal Care Project, The Diabetic Association of Bangladesh, Dhaka, Bangladesh; c The Diabetic Association of Bangladesh, Dhaka, Bangladesh

**Keywords:** mHealth, behaviour change, message development, diabetes, Bangladesh

## Abstract

**Background**: mHealth interventions have huge potential to reach large numbers of people in resource poor settings but have been criticised for lacking theory-driven design and rigorous evaluation. This paper shares the process we developed when developing an awareness raising and behaviour change focused mHealth intervention, through applying behavioural theory to in-depth qualitative research. It addresses an important gap in research regarding the use of theory and formative research to develop an mHealth intervention.

**Objectives**: To develop a theory-driven contextually relevant mHealth intervention aimed at preventing and managing diabetes among the general population in rural Bangladesh.

**Methods**: In-depth formative qualitative research (interviews and focus group discussions) were conducted in rural Faridpur. The data were analysed thematically and enablers and barriers to behaviour change related to lifestyle and the prevention of and management of diabetes were identified. In addition to the COM-B (Capability, Opportunity, Motivation-Behaviour) model of behaviour change we selected the Transtheoretical Domains Framework (TDF) to be applied to the formative research in order to guide the development of the intervention.

**Results**: A six step-process was developed to outline the content of voice messages drawing on in-depth qualitative research and COM-B and TDF models. A table to inform voice messages was developed and acted as a guide to scriptwriters in the production of the messages.

**Conclusions**: In order to respond to the local needs of a community in Bangladesh, a process of formative research, drawing on behavioural theory helped in the development of awareness-raising and behaviour change mHealth messages through helping us to conceptualise and understand behaviour (for example by categorising behaviour into specific domains) and subsequently identify specific behavioural strategies to target the behaviour.

## Background

### mHealth in low- and middle-income countries

The low cost and accessibility of mobile technology means mHealth (the use of mobile technology in health) in low- and middle-income countries (LMICs) has huge potential to reach and improve the health of large numbers of people [1–3]. Due to the nature of technology, mHealth can bypass some of the barriers to health access and knowledge of low-literacy, geographical remoteness and lack of finances [1].

Despite its potential, the evidence base for the effectiveness of mHealth interventions is limited. A review of 76 mHealth studies conducted in LMICs found while there is evidence of effectiveness of some interventions the overall quality and quantity of evidence is limited as many of the studies lack scale and rigorous evaluation [1]. It is possible that poor initial design is a contributing factor to the general lack of scale for mHealth interventions, it may also be that they have not been designed to reach scale. Another review of 16 intervention studies on mHealth covering a range of issues in Africa, Asia and multi-countries found a lack of consistent improvement in behaviour and weak evaluation methods [4]. It did highlight the importance of tailoring messages to an audience, using local language and understanding context to the interventions’ success. While many mHealth behaviour change interventions do not have a clear theoretical framework, a study by Ramachandran and colleagues provides a notable exception [5]. The study was a randomised control trial in India testing the effectiveness of mobile messaging on preventing type two diabetes mellitus (T2DM) among men aged 35–55 with impaired glucose tolerance [5]. The messages were based on the trans-theoretical model of behaviour change and the results indicated a 36% reduction in the incidence of diabetes among this high-risk group over two years [5]. A review of web-based interventions for diabetes management found that having a theoretical base increased the likelihood of success [6]. While there were only nine studies reviewed, most of which were based in high-income countries, their findings support the argument for theory-based approaches to behavioural mHealth interventions. A theoretical base to interventions means the intervention is underpinned and guided by a behavioural model and/or theory of change. Theory-based approaches may be more effective as theories help to explain behaviour and provide a rationale and focus for strategies.

As in other LMICs, despite poor infrastructure and weak health systems, mobile phone use and ownership in Bangladesh is widespread. An estimated 87% of rural households in Bangladesh owned at least one mobile phone in 2013 [7]. While the opportunity for mHealth to promote health has been recognised by NGOs and researchers, a recent scoping study of eHealth and mHealth initiatives in Bangladesh found that they are sporadic and disjointed with a lack of evidence of their effectiveness [8]. UCL and BADAS (the Diabetic Association of Bangladesh) set out to develop an mHealth intervention targeting awareness-raising and behaviour change related to diabetes prevention and control in rural Bangladesh. From the outset we aimed to address some of the gaps in research by ensuring the intervention is contextually relevant, grounded in theory and rigorously evaluated.

### Diabetes in Bangladesh

There were an estimated 422 million adults living with diabetes in 2014 [9], with LMICs accounting for almost 80% of cases [10]. In Bangladesh diabetes affects an estimated 20% to 30% of the adult population either as intermediate hyperglycaemia or fully expressed diabetes mellitus [8]. Ninety percent of diabetes cases are type two diabetes, which is the result of an inadequate production or sensitivity to insulin [10]. Despite the high levels of diabetes, there are low levels of awareness about prevention, control and management of the condition [7] and the resource-poor health system is ill-equipped to meet the demands of the increasing diabetes burden [11].

As part of a three-arm cluster randomised trial [12], we set out to develop, implement and evaluate an mHealth intervention aimed at preventing and managing diabetes among the adult population in rural Bangladesh. Our intervention targeted adults aged over 30, and focused on modifiable risk factors relating to diabetes as recommended by the World Health Organisation [9]: care seeking, diet, physical activity, smoking and stress. The intervention consisted of people signing up (through a community recruitment drive) to receive one-minute voice messages twice a week for 14 months. From the outset we planned for the messages to be informative and entertaining, with professional scriptwriters involved in their production. In order to embed the messages in theory we planned to use the COM-B model of behaviour change [13] to inform the development of the voice messages. In-depth qualitative research in rural Bangladesh ensured the messages were relevant and tailored towards the needs of the message recipients. The content of the messages was therefore informed by both contextual research and the application of behavioural theory, as detailed in the current paper. The intervention developed, informed by this study, was tested as part of the randomised control trial.

## Methods

Development and application of a process for creating content for voice messages was achieved through: 1. Formative research in intervention areas; and 2. Applying theory to formative research findings.

### 1. Formative research

#### Aim

The aim of the formative research was to describe the context of the interventions and inform the development of culturally sensitive, tailored mHealth messages. This included exploration of local understandings of diabetes mellitus and barriers and enablers to having a healthy lifestyle related to specific behaviours (care-seeking, diet, physical activity, smoking and stress).

#### Setting

Data were collected from three *upazillas* (sub-districts) of Faridpur district in central Bangladesh. Faridpur is 200 km^2^ with a population of 1.7 million. Farming (jute and rice) is the main livelihood source in the district. The population is mostly Bengali and 90% Muslim [14]. Data from our trial baseline survey in the area reveal approximately 10% of the population have diabetes and 20% have intermediate hyperglycaemia [15].

#### Sampling and data collection

In total 16 semi-structured interviews and nine focus group discussions (FGDs) were conducted. Six diabetics (3 women, 3 men), 5 non-diabetics (3 women, 2 men) and 5 health care workers were interviewed. Focus group discussions were conducted with five groups of diabetics (3 women, 2 men) and 4 groups of non-diabetics (2 women, 2 men). Research respondents were purposively sampled. They were recruited through key informants, snowball sampling and the assistance of local staff from the Diabetic Association. The respondents were aged between 30 and 60. The researcher sought to achieve a sample in which approximately half were perceived to be overweight, and there was a balance of better off and poorer socio-economic groups as estimated by observing house construction materials. This provided a range of views that aimed to be reflective of the rural population. Additionally, five local health workers were recruited in order to triangulate findings.

The interviews and FGDs were conducted in Bengali by a qualitative, bilingual researcher from BADAS (KAk). The interviews followed a topic guide, which is a list of topics and open-ended questions that serve as a guide for the interviewer. The topic guides were developed on the basis of the aims of the research, literature reviews and COM-B theory of behaviour change. The topic guides were developed in English, translated into Bangla and piloted with two non-diabetic participants in a suburb of Dhaka and one health worker and one person with diabetes at BIRDEM (Bangladesh Institute of Research and Rehabilitation in Diabetes Endocrine and Metabolic disorders) hospital in Dhaka. In addition to the interview schedule, mapping of the village and pile sorting (pictures described and categorised by research participants) were used in the interviews and FGDs in order to promote discussion.

#### Data analysis

The FGDs and interviews were recorded and transcribed from Bangla into English by professional translators. The translations were checked and parts back-translated to ensure accuracy. The data were analysed by two UCL researchers (JM and HMJ) and one from the BADAS (KAk). The software NVIVO 13 was used to assist, share and organise the analysis. Descriptive content analysis [16] was used. Transcripts were analysed thematically. The process involved the researchers who analysed the data (JM, HMJ and KAk) familiarising themselves with the data, independently listing emerging themes (patterns in the data), comparing notes and reassessing the themes and data [17]. The data and discussed themes were presented to the wider trial team (all the researchers involved in the randomised control trial) before finalising the coding structure and coding the transcripts in NVivo.

These data were subsequently organised and tabulated according to barriers (things that prevent) and enablers (things that assist) healthy behaviours that the intervention focuses on – general cross-cutting themes, care-seeking, diet, physical activity, smoking and stress. The result was a detailed list of barriers and enablers to a healthy lifestyle for each focus area, complete with quotes and context.

### 2. Applying theory to formative research to inform content

#### Selecting a theory

The COM-B model [13] was referred to in the original project proposal as the framework we would use to develop and guide evaluation of the intervention. COM-B and its corresponding ‘behaviour change wheel’ (BCW) is an integrated framework based around a ‘behaviour system’ known as COM-B: Capability, Opportunity, Motivation-Behaviour [13], that explains behaviour and what needs to be addressed in order for behaviour to change. Capability is the psychological and physical capacity to engage behaviour. Motivation is defined as processes that energise and direct behaviour. Opportunity is factors outside the individual that make the behaviour possible. The model is broad as it was developed from 19 existing frameworks of behaviour change [13]. The comprehensiveness of the model has been criticised, as by synthesising such a range of approaches it means that complex theories have been simplified and it is difficult to unpack exactly what is effective [18,19]. However, given the heterogeneity of the target of our intervention (variety of ages, gender, socio-economic status and health needs) it was difficult to assume a single process or model will be applicable for all as focused behaviour-change models tend to rely on specific processes working within limited domains [19]. Furthermore, in practice intervention design frequently draws on several behaviour theories with overlapping theoretical constructs which makes it difficult to identify the exact process underlying behaviour change [20]. So, while we did look at other more specific models, we decided the broadness of COM-B made it more suitable for our context.

Corresponding to COM-B, and further elaborating it, the Theoretical Domains Framework (TDF) was developed [20] and thus also considered for application in our project. TDF is an integrative framework of behaviour change theory that simplifies and integrates existing theories to make them more accessible [20]. TDF was developed through consensus by a range of experts, and later refined and validated by specialists [20,21]. TDF covers 14 domains of theoretical constructs that are a useful way of understanding and classifying behaviour. Examples of the domains include knowledge, skills, social influences, beliefs about capabilities, social influences, and environmental context and resources. Additionally, specific behaviour change techniques (BCTs) have been identified to correspond with individual domains [20]. BCTs are the smallest constituents of behaviour change interventions; they are both replicable and observable [22]. Examples of BCTs include shaping knowledge, modelling behaviour, information about health consequences and goal setting. A BCT taxonomy consisting of 93 BCTs has been created through a series of consensus exercises involving over 50 behaviour change experts [20]. While the individual BCTs have been critiqued as being too simple and overly prescriptive [18], the range allows choice and it would be difficult to apply overly complex BCTs to our mHealth intervention due to the constraints of short voice messages.

As our intervention covers a broad population we needed an understandable theory that comprehensively covers behaviour, thus we utilised both COM-B and TDF frameworks. COM-B had framed much of our formative research and was easier to communicate with the wider research team. COM-B was utilised in association with TDF in helping to identify TDF components that are likely to be important in changing behaviour [20,23]. The TDF model further elaborated COM-B and was a tool suited to the practical application of a range of behaviour change techniques in our study population, and thus was used as a tool to specifically guide the messages.

#### Applying TDF and COM-B to formative research to inform content

A paper by French and colleagues in 2012 outlined practical steps to developing an intervention by considering theory, evidence and practice [24]. We drew on this approach when developing our intervention. Table 1 summarises French et al’s model and identifies elements we drew on. This included the need to specify the behaviour change we are targeting, identifying barriers and enablers that need to be addressed and applying appropriate BCTs. However, we tailored our approach to specifically address TDF for an mHealth intervention, meaning we added, omitted and adapted steps. For example, step 2 of French et al’s model was broken down and adapted to align with TDF, we omitted step 4 from French et al’s model and we added our own steps 1 (context of the intervention) and 6 (a table of content bringing together the earlier steps).

**Table 1.** Steps for developing a theory informed implementation intervention: summary of French et al (2012) and mHealth intervention content development.

Step	Tasks (summarised)	mHealth intervention
STEP 1: Who needs to do what, differently?	Identify the evidence-practice gap Specify the behaviour change needed	The specific outcomes and areas of behaviour change were identified
STEP 2: Using a theoretical framework, which barriers and enablers need to be addressed?	Select which theory(ies)/theoretical framework(s) are likely to inform the pathways of change Use the chosen theory/framework, to identify possible barriers and enablers to that pathway Use qualitative and/or quantitative methods to identify barriers and enablers to behaviour change	TDF and COM-B were selected. Barriers and enablers to behaviour change identified through qualitative formative research Barriers and enablers categorised in terms of TDF
STEP 3: Which intervention components could overcome the barriers and enhance the enablers?	Use the chosen theory/framework, to identify potential BCTs to overcome the barriers and enhance the enablers Identify evidence to inform the BCTs Identify what is likely to be feasible, locally relevant, and acceptable	BCTs identified according to specific enabler and barrier domains
STEP 4: How can behaviour change be measured and understood?	Identify mediators of change to investigate the proposed pathways of change Select appropriate outcome measures and determine their feasibility	Through the process evaluation and cluster randomised controlled trial design the mechanisms of change will be evaluated. This is not directly part of the message development.

For our intervention development we considered the outcomes needed for the mHealth intervention to be a success and we were able to identify the barriers and enablers to this through the formative research. TDF theory enabled us to systematically classify the barriers and enablers and thereby identify BCTs to address them. We were able to break down this process into six-steps as detailed in the results.

## Results

Through the analysis of the qualitative research and the TDF framework, a six-step process to developing a guide for the content of behaviour-orientated voice messages was produced. The end result was a comprehensive guide for the study team as well as scriptwriters and producers of the voice messages (who come from a non-medical, non-academic or behaviour change background). Table 2 outlines the steps, with more detail provided under the corresponding sub-headings below.

**Table 2.** Steps to message content development.

Step	Summary of step
STEP 1: Context of the intervention	The formative research provides an in-depth analysis of the context of the intervention.
STEP 2: Break down intended outcomes	Related to the formative research and the overall outcomes of the project, specific outcomes for the five areas of focus, i.e. care-seeking, diet, physical activity, smoking and stress, were identified.
STEP 3: Identify and list the enablers and barriers to behaviour change	Enablers to promoting a healthy lifestyle and barriers to implementing a healthy lifestyle were identified from the formative research and listed.
STEP 4: Categorise the barriers and enablers according to COM-B and the TDF	The identified enablers and barriers were categorised according to TDF and COM-B.
STEP 5: Suggest behaviour change approaches for each enabler and barrier	In light of the appropriate transtheoretical domains, behaviour change approaches were identified for each enabler and barrier
STEP 6: Table of message content produced based on the intended outcomes, barriers and enablers and BCTs	A table of message was produced based on the intended outcomes and assigned BCTs addressing each enabler and barrier.

### Step 1: the context of the intervention

An overview and key findings from the formative research were shared with those involved in message development. A full description is beyond the scope of this paper, instead we provide a summary of some of the key findings on context that directly influenced mHealth message development, in Table 3, with specific emphasis on themes of religion, balance, family and societal pressure and gender roles. There were aspects on which the messages were able to build on, for example the responsibility to look after oneself as a religious duty. Importantly, understanding of context was crucial to defining the behaviour the intervention aimed to influence in step 2.

**Table 3.** Context from formative research.

Aspect	Description	Influence on the messages
Religion and belief	The importance of religion and a belief that everything is under the control of *Allah* was crucial in peoples’ understanding of their health. While many still valued medical advice, this belief could lead to people being fatalistic about their health and less motivated to change behaviour as explained by one respondent ‘*Allah has given us this disease…It’s not about being rich or being poor. Who has bad luck will have diabetes no matter what they do’* (diabetic woman, FGD021). However, some people also spoke about how because Allah gave life it is one’s responsibility to look after it. Additionally during Ramadan ‘bad habits’ and ‘unhealthy’ behaviour (such as smoking) reduced.	Emphasis on responsibility to look after ones’ health. Additional messages were created to correspond with the month of *Ramadan* and how diet should be approached
Balance	Routines, balance and moderation were perceived as key to achieving health; eating regularly and reasonable portion sizes, getting enough rest and work are examples of balance. One respondent explained ‘*Maintaining three proper meals every day is enough to keep us healthy. Regular eating, bathing, and proper lifestyle – that is enough’* (diabetic man, FGD023).	This was built on – the need for regular and reasonable sized meals was emphasised
Family and social pressure	Whether family members valued and supported each other affected an individual’s access to treatment and their welfare; for example women often rely on their husband to take them to the doctor, and the family diet depends on what the mother has prepared. Social norms are important factors in affecting one’s behaviour. For example, hospitality is very important with people expected to serve and consume different foods during visits and on special occasions, as explained by a respondent ‘*In a social ritual…or in a gathering, if I refuse the dishes offered to me it would not be polite.’* (diabetic woman SSI008).	Messages targeted the whole family. Specific examples in social situations were drawn on/highlighted
Gender	Social norms are highly gendered as women are expected to behave in a certain way and are judged accordingly. Seclusion prevents some women from going outside of the home making it difficult for them to walk or be physically active. One health worker explains *‘walking is hardly possible for most of the women in the village for some reasons. Where should they walk?’*(health worker SSI020). Additionally there are strict gender roles within society and families – for example men do most of the food shopping and women prepare and cook.	Messages were tailored to men and women, they also highlighted the importance of women being able to engage in ‘healthy’ behaviour.

### Step 2: breakdown of outcomes

When planning an intervention it is important to identify changes the intervention should have (i.e. outcomes). The overall primary outcome of the trial was the reduction in the prevalence of intermediate hyperglycaemia and T2DM and a decrease in the two-year cumulative incidence of T2DM among individuals with intermediate hyperglycaemia [12]. Secondary and explanatory trial outcomes include a range of outcomes related to risk factors, awareness and control of diabetes.

**Figure 1.**
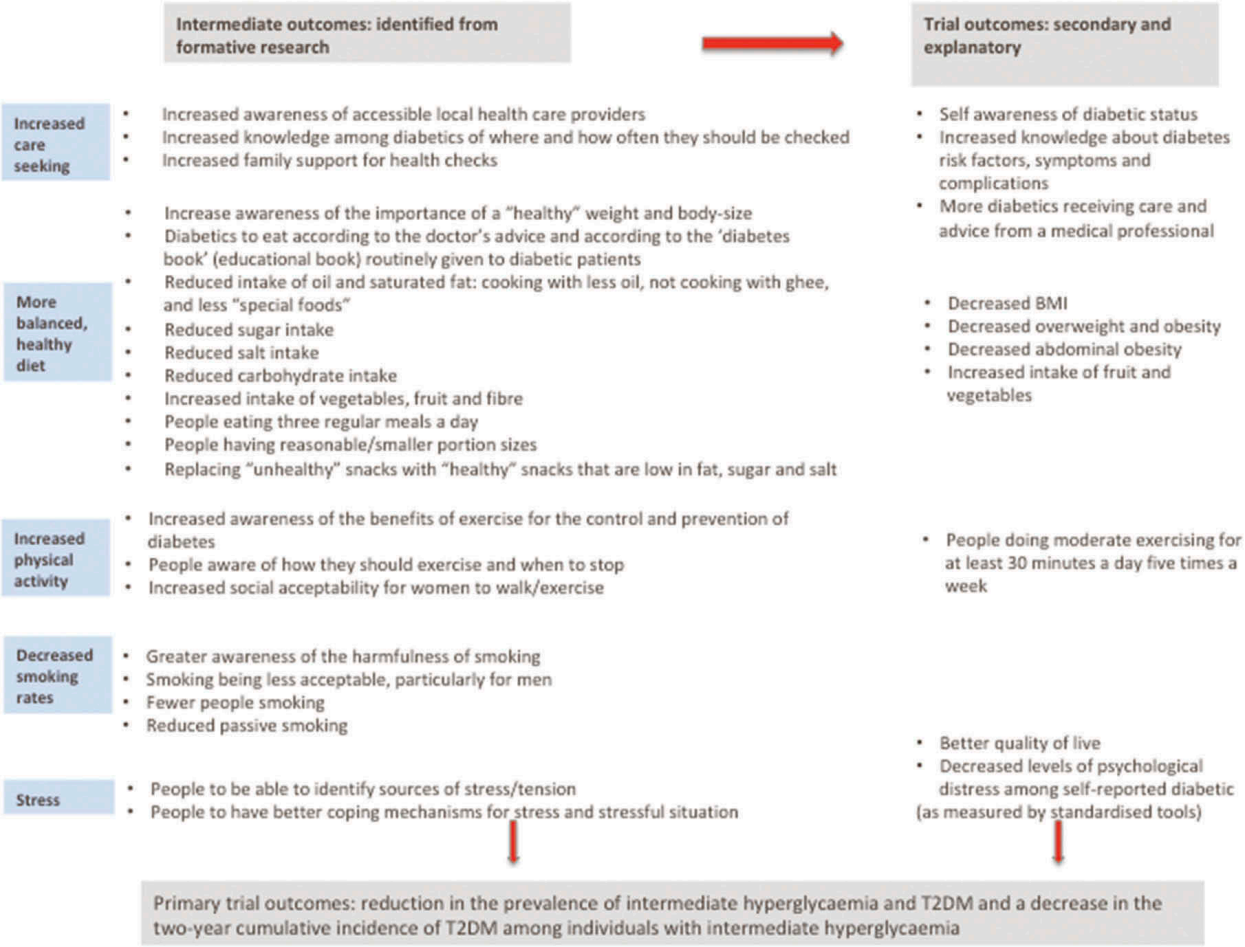
List of intended outcomes for the intermediate and trial outcomes.

We developed a comprehensive list of intended intermediate outcomes for the intervention focused on behaviour and awareness, and related to each of our focus areas (Figure 1). The intermediate outcomes are behaviours that need to change in order to achieve the trial outcomes, and are directly relevant to the context of the intervention and emerged from the formative research as well as the secondary trial outcomes (reported in full elsewhere [12]). Having a clear understanding of the intended consequences of the intervention helped focus the messages of the intervention as well as identify the barriers and enablers to achieving them.

### Steps 3: identifying and listing enablers and barriers to behaviour change

As explained in the methods section, the analysis of the formative research included a detailed breakdown of the barriers and enablers according to areas of focus. This list of barriers and enablers provided the basis of the message content, and enabled targeting of behaviour change specific to the context and grounded in theory. Table 4 provides examples of barriers and enablers from each focus area.

**Table 4.** Examples of barriers and enablers to a healthy lifestyle (from formative research)^a^.

Enablers	Barriers
**General**	
Allah gave you life it’s your responsibility to keep it healthy	Fate determines diabetes/health status
**Care-seeking**	
Diabetics tend to go for check-ups/testing if they feel unwell	Lack of consistency in taking medicines: patients may decide themselves that they feel better and stop taking medication
**Diet**	
Family support and encouragement to change eating habits i.e. all eating *ruti* in the evenings, mother cooking ‘healthy’ foods, daughter encourages grandmother to eat with less salt etc.	Family not changing or supporting different eating habits i.e. husband demanding food is cooked with more oil, wife not changing cooking practices
**Physical activity**	
Can integrate walking into routine (walking children to school, going to the shops, walking to work etc.)	Social acceptability: Not always socially acceptable for women to be walking outside and judgements made
**Smoking**	
Knowledge: greater public awareness of health and smoking i.e. warnings of cigarette packages and doctor’s advice	Knowledge: overall general unawareness about the harmfulness of smoking to health No awareness regarding smoking and the link to diabetes
**Stress**	
Some coping mechanisms identified: talking to someone, music, religious rituals etc.	‘Unhealthy’ coping mechanisms: smoking, taking too many or unnecessary pills

^a^There are some empty cells in this tables. This is because where possible we match barriers to enablers. If there is not a matching enabler or barrier we leave the corresponding cell blank.

### Steps 4 and 5: dividing the enablers and barriers according to TDF and COM-B and suggesting behaviour change techniques for the messages

For each enabler and barrier a COM-B characteristic and transtheoretical domain was identified. Identifying underlying domains enabled a better understanding of the behaviour and appropriate BCTs associated with the domains could be identified. Drawing on the BCT taxonomy compiled by the same group who developed TDF [22], BCTs were selected for each enabler and barrier. BCTs that could be selected were limited due to the nature of voice messages. Identified BCTs included: modelling (demonstrating) behaviour, shaping knowledge, information about consequences, repetition and substitution, social support (encouraging), pros and cons and goal setting. Table 5 provides examples of TDF and BCTs for a selection of barriers and enablers (the complete table of TDF and BCTs identified for each barrier and enabler are shown in Table 6). The completed table enabled us to align messages with each barrier and enabler, and ensure that they were all addressed.

**Table 5.** Examples of barriers and enablers to a healthy lifestyle divided by COM-B and TDF domains, and associated Behaviour Change Techniques.

TDF Domain/ COM-B	Enabler	Barrier	Behaviour Change Technique
TDF: 1. Knowledge COM-B: Capability	Greater public awareness of the link between smoking and ill health	Overall lack of awareness regarding the link between smoking and ill health, particularly diabetes and smoking	Shaping knowledge Information about consequences
TDF: 2. Skills COM-B: Capability	Some coping mechanisms identified: talking to someone, music, religious rituals etc.	Lack of control and coping mechanisms	Shaping knowledge: identify stress, look for coping strategies Modelling behaviour
TDF: 6. Beliefs about consequences COM-B: Motivation	Religious beliefs and responsibility	Religious beliefs and fate	Information about consequences Shaping knowledge: personal responsibility and reinforcing the enabler
TDF: 11. Environmental context and resources COM-B: Opportunity	Walking with other people	Women feel unsafe walking alone	Modelling behaviour: examples of people walking together
TDF: 12. Social influences COM-B: Motivation	Family supporting different/healthy eating habits	Cooking: women cooking with high levels of oil etc., men asking for it	Modelling behaviour Shaping knowledge

**Table 6.** Complete list of barriers and enablers to a healthy lifestyle divided by COM-B and TDF domains, and associated Behaviour Change Techniques.

General			
TDF domain/ COM-B	Enabler	Barriers	Behaviour change technique
TDF: 1. Knowledge COM-B: Capability	People have some knowledge about diabetes and its management	Lack of in-depth knowledge about the causes of diabetes	Shaping knowledge: build on enablers
	Some knowledge that sedentary lifestyle causes diabetes		Shaping knowledge
	Some knowledge about hereditary nature of high blood pressure and connection between high BP and diabetes	Belief that diabetes is contagious	Shaping knowledge: challenge incorrect beliefs
		Lack of knowledge about how to prevent diabetes	Shaping knowledge
TDF: 4. Beliefs about capabilities COM-B: Motivation		Beliefs: too many pills can make one unwell, older people put on weight, complications other than diabetes blamed for making one feel unwell	Shaping knowledge Information about health consequences
		Feelings of lack of control over body weight, health and diabetes	Modelling behaviour Goal setting Information about health consequences
		Difficulties to convince pre-diabetics to change	Modelling behaviour Goal setting
TDF: 6. Beliefs about consequences COM-B: Motivation	Religious beliefs and responsibility	Religious beliefs and fate	Information about health consequences Shaping knowledge
	Bad habits stopped during Ramadan		Modelling behaviour: encourage this to continue
	Routine, balance and moderation = healthy lifestyle		Modelling behaviour
		People not taking responsibility for their health	Information about health consequences
TDF: 10. Memory, attention and decision making COM-B: Capability		Difficulties to maintain a routine	Modelling behaviour Social support (encourage)
		People identified as being ‘careless’	Modelling behaviour, Social support (encourage)
		Perception that if you are addicted there is nothing that can be done (smoking, sugar etc.)	Modelling behaviour
TDF: 11. Environmental context and resources COM-B: Opportunity	Lifestyle changes are not too complicated and within peoples’ reach	Poverty makes it difficult to maintain a moderate, regular lifestyle	Modelling behaviour
		Poverty and time constraints make it difficult to manage/control diabetes	Modelling behaviour
		Increase in stress = increased BP and poor health	Social support (encourage)
TDF: 12. Social influences COM-B: Opportunity	‘Slim’ perceived as being healthy	Fat looking good	Shaping knowledge: challenge perception
	Diabetes thought to damage appearance		Shaping knowledge
	Advice and criticism from friends	Criticism from friends	Social support (encourage)
	Family support for management of diabetes		Social support (encourage) Modelling behaviour
TDF: 13. Emotion COM-B: Motivation	Good explanations of diabetes can reduce fear	Diabetes and complications cause fear	Shaping knowledge

Care Seeking			
TDF Domain/ COM-B	Enabler	Barrier	Behaviour Change Technique
TDF: 1. Knowledge COM-B: Capability	Doctors offering advice on lifestyle improvement factors	Lack of awareness on how to prevent diabetes	Shaping knowledge
TDF: 3. Social/Professional Role and Identity COM-B: Motivation	Women and poor people go for regular check-ups	Better-off don’t think check-ups are so important	Shaping knowledge: everyone needs to go to the doctor Modelling behaviour
TDF: 4. Beliefs about capabilities COM-B: Motivation		Belief in fate and a lack of control to seek care	Shaping knowledge Information about health consequences Modelling behaviour
	Testing own blood sugar is empowering and motivates a person to control their diabetes		Pros and cons Shaping knowledge
	Taking medicinal plants makes someone feel in control of their diabetes	Medicinal plants unregulated and could be safety concerns	Pros and cons Shaping knowledge
TDF: 6. Beliefs about consequences COM-B: Motivation	Diabetics will go for check-ups if they feel unwell	Not taking medicines because they are not improving or because they improve feel they no longer need to take them	Modelling behaviour Shaping knowledge Information about health consequences
		Waiting until diabetes is ‘bad’ or suffering from complications before seeking care	Shaping knowledge Information about health consequences
		Belief that medication is enough to treat diabetes, without lifestyle changes	Shaping knowledge Modelling behaviour
TDF: 10. Memory, attention and decision process COM-B: Capability		Forgetting to take medication, particularly when not in a routine	Habit formation: suggest a reminder Imaginary reward
		Descriptions of being too ‘lazy’ and ‘careless’ to take medicine	Habit formation Shaping knowledge
		Diabetes book (provided by healthcare providers) difficult to understand	Shaping knowledge: providing straight forward information
TDF: 11. Environmental context and resources COM-B: Opportunity	Some people request local pharmacy to carry medicine	Strips, insulin etc. not always available locally	Modelling behaviour
	Examples of high quality of care	Low quality of care, chaotic treatment; having to wait/crowds	Modelling behaviour: Acknowledge difficulties and suggest ways of overcoming
	Dr’s consulting specialists by phone, specialists visiting villages once a month	Lack of training and resources to treat diabetes locally	Modelling behaviour: Pros and cons
	Free services will motivate people to seek care	Costs: travel, tests, check-ups, medicine	Shaping knowledge: importance of check-ups Pros and cons
	Doctors prescribing locally		Shaping knowledge
		Local services can’t confirm a diagnosis of diabetes – will refer to specialists/Faridpur	Modelling behaviour: Pros and cons
		Herbs taken due to costs of medicines	Shaping knowledge Pros and cons
		Business/lack of time to take medicine and visit facilities: particularly for women	Modelling behaviour: examples of balancing and prioritising Pros and cons
TDF: 12. Social influences COM-B: Opportunity	Family support: taking to health facilities, arranging appointments, encouraging to seek care	Lack of family support: women rely on husbands to get strips and to take them to the doctor	Modelling behaviour: examples of how can support family
		Women not feeling comfortable talking about health/sensitive issues	Modelling behaviour
TDF: 13. Emotion COM-B: Motivation	People reporting understanding a doctors’ advice	Fear of doctors	Shaping knowledge
	Trust, rapport with a doctor		Modelling behaviour Pros and cons
	Fear of dying can mean people take advice seriously		Shaping knowledge
		Fear after diagnosis prevents patients coming back for care/check-ups	Shaping knowledge: stress diabetes is manageable if controlled
		Feeling out of control	Shaping knowledge:
TDF: 14. Behavioural regulation COM-B: Capability	If treatment is planned in stages patients more likely to return and not feel overwhelmed		Modelling behaviour Goal setting: encourage people to have targets

Diet			
TDF Domain/ COM-B	Enabler	Barrier	Behaviour Change Technique
TDF: 1. Knowledge COM-B: Capability	Basic knowledge about a diabetic diet	Lack of in-depth knowledge/knowledge on portions	Shaping knowledge
	Basic knowledge about ‘good’/’bad’ food	Lack of in-depth knowledge, confusion, incorrect knowledge	Shaping knowledge
	Desire for more knowledge		Shaping knowledge
	Dr’s advice valued and people report trying to follow it		
		Lack of knowledge about diet and prevention of diabetes	Shaping knowledge
		General lack of understanding about the seriousness of diabetes	Shaping knowledge
TDF: 2. Skills COM-B: Capability	Growing vegetables/home gardens		Modelling behaviour
TDF: 4. Beliefs about capabilities COM-B: Motivation		Lack of control: belief will put on weight despite what one eats, concept of ‘body letting me down’	Shaping knowledge
TDF: 6. Beliefs about consequences COM-B: Motivation	Allah gave life and our responsibility to look after it	Religious beliefs and fate – changing eating habits will not help	Information about health consequences Shaping knowledge
	‘Home-cooked’ food believed to be healthy and ‘outside’ food unhealthy		Shaping knowledge: building on existing knowledge
	Balance in food considered to be good		Shaping knowledge
	Diagnosis of diabetes encouraging to change eating habits		Shaping knowledge Information about health consequences
	Border-line/people at risk of diabetes will try to follow doctor’s advice		Shaping knowledge Information about health consequences: building on existing motivation
		Feeling better after changing diet/medication means diabetics may revert to old habits as believe they are ‘better’	Shaping knowledge Modelling behaviour
		Belief that non-diabetics can eat whatever they like	Shaping knowledge
TDF: 8. Intentions COM-B: Motivation	Personal motivation to eat well and refuse certain foods		Modelling behaviour
	Good practices: making snacks with reduced sugar, replacement sugars in tea, ‘raw tea’ drinking		Modelling behaviour
TDF: 10. Memory, attention and decision process COM-B: Capability	Good practice: developing the habit of eating with less salt and sugar		Modelling behaviour
		Habit of snacking inside and outside the home	Shaping knowledge
TDF: 11. Environmental context and resources COM-B: Opportunity	Cost of food: *daal* and vegetables reasonable price	Cost of food: *ruti*, eggs, meat, fruit more expensive	Shaping knowledge Modelling behaviour
	Education: means people are more likely to follow ‘rules an regulations’	Lack of education	Shaping knowledge
	Booklet provided by some care providers explaining what food and portions diabetics should eat found useful	Lack of availability of this booklet and other resources	Shaping knowledge: increase awareness of available resources
		Underweight and malnourishment a problem	Shaping knowledge: giving practical advice that considers a range of people
		Lack of time to eat regularly	Modelling behaviour
		Lack of time to cater to everyone’s nutritional needs	Modelling behaviour Pros and cons
		Availability of ‘unhealthy’ food inside and outside the home	Modelling behaviour Pros and cons
		Convenience of eating outside the home	Modelling behaviour Pros and cons
		Fertilisers, chemicals etc. used to grow food	Shaping knowledge Pros and cons
TDF: 12. Social influences COM-B: Opportunity	Body image: Being ‘slim’ perceived as healthy (not too thin, not too fat)	Body image: being ‘heavier’ perceived as healthy and beautiful	Shaping knowledge: changing/reinforcing perceptions
	Body image: extra fat meaning there are more diseases, can cause difficulties		Shaping knowledge
	Cooking: women may cook with lower levels of oil etc.	Cooking: women cooking with high levels of oil etc. as men (husbands, fathers, in-laws etc.) are asking for it	Modelling behaviour Shaping knowledge: whole family affected by cooking
	Family supporting different/healthy eating habits	Family not supporting different/healthy eating habits	Modelling behaviour Pros and cons
	Shopping: men shop, women can intervene		Modelling behaviour
	Good practices: family and friends bringing/serving alternatives to sweets/snacks	Hospitality: expected to eat and serve foods during social occasions and visits	Modelling behaviour
		Social gatherings and meeting in tea shops	Modelling behaviour
		Status and food: eating meat, *ghee* etc. can be associated with being a higher social status	Shaping knowledge Pros and cons
TDF: 13. Emotion COM-B: Motivation	Feeling unwell when eating unhealthy food	Feeling unwell, hungry, having gas etc. when having smaller portions/healthy food	Shaping knowledge: stress long-term benefits
	Diabetics feeling better when eating healthy food		Shaping knowledge Modelling behaviour
	Eating less and better during Ramadan		Shaping knowledge Goals setting: suggest continuing some of the behaviour after Ramadan
		Taste and enjoyment of certain foods that are unhealthy	Information about health consequences Pros and cons Shaping knowledge: promoting moderation Modelling behaviour: cooking tasty, healthy food
		Lack of concern for health and living for ‘now’	Information about health consequences Pros and cons Shaping knowledge:
		Importance of rice: complete meal, nourishment etc.	Information about health consequences Pros and cons Shaping knowledge

Physical Activity			
TDF Domain/ COM-B	Enablers	Barrier	Behaviour Change Technique
TDF: 1. Knowledge COM-B: Capability	Some knowledge exercise is good for diabetics	Not a detailed knowledge of the relationship between exercise and diabetes	Shaping knowledge
	Some awareness exercise is related to body weight		Shaping knowledge
	Doctors advice that walking helps the body to create its own insulin		Shaping knowledge: reinforce/build on this knowledge
	Diabetics understand/take doctors’ advice		Shaping knowledge Modelling behaviour
		Lack of knowledge that exercise can help prevent diabetes	Shaping knowledge Information about health consequences
TDF: 2. Skills COM-B: Capability	Men: Swim, do push-ups, walk, some sports Women: walk, stretch, occasionally swim		Shaping knowledge Modelling behaviour
TDF: 3. Professional role and identity COM-B: Motivation	People who exercise seen as educated		Modelling behaviour: exercise is for everyone
		Exercise is seen as a sign of having diabetes/done by ‘fat’ people	Shaping knowledge Modelling behaviour
TDF: 4. Beliefs about capabilities COM-B: Motivation		Having diabetes makes people feel unwell, therefore difficult to do exercise	Shaping knowledge: exercise makes people feel better in the long-term Goal setting
TDF: 6. Beliefs about consequences COM-B: Motivation		Unsure/unconvinced about the benefits of exercise:	Shaping knowledge
		Belief hard work is enough to keep healthy, there is no need to do other exercise	Shaping knowledge
TDF: 10. Memory, attention and decision making processes COM-B: Capability		No habit of walking (availability of cheap transport)	Goal setting Repetition and substitution: habit formation
TDF: 11. Environmental context and resources COM-B: Opportunity	Able to integrate walking into everyday routine	Lack of time to exercise/walk	Shaping knowledge: Modelling behaviour
	Walking with other people	Women feel unsafe walking alone	Modelling behaviour:
	Rural areas do have more open spaces than urban areas	Lack of space/places to exercise	Shaping knowledge: types of exercise that are possible
		Weather/muddy roads make it difficult to walk	Pros and cons
		Other people do household works (women, servants, younger people), therefore others are less active	Shaping knowledge
TDF: 12. Social influences COM-B: Opportunity	Friends recommending to walk to manage diabetes		Modelling behaviour
	Walking with friends feels good, encourages walking		Modelling behaviour
	Not walking viewed as ‘lazy’		Modelling behaviour (Need to be careful not to stigmatise people)
		Social acceptability: sports not seen as socially acceptable for older people or women	Shaping knowledge: importance of exercise and challenge perceptions Social support: encourage support
		Women feel judged/shamed if walking around outside (especially if they get muddy etc.)	Social support Pros and cons
		Exercise viewed as not a normal thing to do	Social support Pros and cons
		Exercise viewed as only for those in the city who have no manual labour	Social support Pros and cons
TDF: 13. Emotion COM-B: Motivation	Feeling better/good after manual work/exercise		Shaping knowledge Information about consequences
		Fear of getting injured when playing *ha dudu*; risk of getting cold after swimming	Pros and cons

Smoking			
TDF Domain/COM-B	Enablers	Barrier	Behaviour Change Technique
TDF: 1. Knowledge COM-B: Capability	Greater public awareness of the link between smoking and ill health	Overall lack of awareness regarding the link between smoking and ill health. No awareness of the link between smoking and diabetes	Shaping knowledge Information about health consequences
	Dr’s advise to give up smoking		Shaping knowledge: reinforce doctor’s advice
TDF: 4. Beliefs about capabilities COM-B: Capability		Belief that can only give up by quitting completely	Shaping knowledge: about how to reduce gradually Goal setting
TDF: 6. Beliefs about consequences COM-B: Motivation	People quit due to physical health problems	People wait to quit until they have physical health problems	Shaping knowledge Information about health consequences
TDF: 8. Intentions COM-B: Motivation	Personal motivation to stop smoking	People still smoke despite doctor’s advise	Pros and cons Information about health consequences
TDF: 10. Memory, attention and decision process COM-B: Capability		Addiction to smoking	Goal setting Pros and cons
TDF: 11. Environmental context and resources COM-B: Opportunity TDF: 12. Social influences COM-B: Motivation	Economic costs discourages from smoking	People smoke to suppress hunger	Pros and cons
	Stigma: generally not accepted for women to smoke	Very normal for men to smoke	Shaping knowledge Social support
	Stigma: when smoking in front of elders, women etc.		Shaping knowledge: information on the effects of passive smoking
	Not acceptable to smoke in public spaces (bus, mosques etc)		Shaping knowledge Information about health consequences
	Family: less likely to smoke if it is not done in the family		Modelling behaviour
	Family: discouraging smoking/encouraging to give up		Modelling behaviour Social support (encouraging)
	Religion discouraging smoking, people giving up for religious reasons		Modelling behaviour: build on this motivation
	Quitting smoking because of work		Shaping knowledge: reasons to quit Pros and cons
	Less likely to smoke with age		Shaping knowledge:
		Smoking perceived to be common among certain groups: farmers, younger people, people in rural areas, people in university	Shaping knowledge: on the extent of problem Social support: encouraging people to quit Modelling behaviour
		Introduction at a young age to smoking by others	Modelling behaviour: example of someone introduced to smoking and later regretting it Information about health consequences
		Peer pressure to smoke	Modelling behaviour: examples of peer pressure
		Smoking is a social activity	Modelling behaviour Pros and cons
		Incentives to smoke: as part of a political campaign	Modelling behaviour Pros and cons
		Men and women also take other tobacco products	Shaping knowledge: information about other tobacco products
TDF: 13. Emotion COM-B: Motivation		Pleasure and comfort of smoking	Pros and cons: acknowledge comforts of smoking, but also the negatives
		Smoking relieves stress	Pros and cons Modelling behaviour: alternative ways to deal with stress

Stress			
TDF Domain/ COM-B	Enablers	Barrier	Behaviour Change Technique
TDF: 1. Knowledge COM-B: Capability	Some knowledge that stress can make diabetes worse	Most people did not link stress and diabetes	Shaping knowledge: the link between diabetes and stress
	Some understanding that stress affects health		Shaping knowledge
TDF: 2. Skills COM-B: Capability	Some coping mechanisms identified: talking to someone, music, religious rituals etc. (See more below)		Modelling behaviour
TDF: 4. Beliefs about capabilities COM-B: Motivation		Lack of control and coping mechanisms	Shaping knowledge: identify stress, look for coping strategies, acknowledge some things are not within the individuals’ control Modelling behaviour
TDF: 10. Memory, attention and decision making process TDF: Capability	Coping mechanisms: music, watching TV, reading		Modelling behaviour Pros and cons: of different coping mechanisms, stress finding the right ones
	Coping mechanisms: distraction, focusing on other things		Modelling behaviour Pros and cons
		‘Unhealthy’ coping mechanisms: smoking, taking pills	Pros and cons
TDF: 11. Environmental context and resources COM-B: Opportunity	Identified sources of pleasure: money, security, health	Identified sources of stress: money, poverty, land	Shaping knowledge
	Health professionals able to treat the symptoms of stress: hypertension, headaches etc.	Not dealing with the root causes of stress	Shaping knowledge Pros and cons
TDF: 12. Social influences COM-B: Opportunity	Sources of pleasure: family, socialising	Sources of tension: family, responsibilities, early marriage, conflict in family	Shaping knowledge
	Coping mechanisms: talking to others	Others will know their problems if they talk about them	Social support (encourage) Modelling behaviour
	People of the village come together to help those in need e.g. if sick		Social support (encourage) Modelling behaviour
TDF: 13. Emotions COM-B: Motivation	Coping mechanisms: music, praying, rituals, being alone, resting		Modelling behaviour Pros and cons
		Symptoms of stress: poor health, headaches etc.	Shaping knowledge

**Additional notes**

In the working table of content for script writers there was an additional column entitled ‘message number’ – this way we were able to add the message numbers that addressed the individual barriers and enablers – allowing us to track the messages and ensure all the barriers and enablers were addressed.

In the final column of this table ‘behaviour change technique’ some additional information explaining how the BCT can be approached is occasionally added – again there was more information in the original table.

The BCT ‘modelling behaviour’ refers to ‘demonstration of the behaviour’ in the BCT taxonomy.

### Step 6: producing table of message content based on the intended outcomes, barriers and enablers and BCTs

In order to guide the scriptwriters as to the content of the voice messages we created a table with the guidelines for the content of individual messages. The details in the table include the TDF, BCT, barriers/enablers, the content, audience and suggested format. Each message was based around a specific enabler and/or barrier. The content addresses the barrier and/or enablers through one of the BCTs suggested.

An example of one of the messages is in Table 7. This message addresses the barrier that people are often expected to eat sweets and rich food during social occasions. The BCT is modelling behaviour: hence a drama with a scenario of someone going to a wedding and the techniques someone uses to eat smaller portions and less sweet food is described. The scenario was informed by the outcomes (smaller portions, less sugar, less oil etc.) and the context (the types of food and events extracted from the data). Doctors working with diabetic patients in Bangladesh checked all the messages to ensure they are in-line with current medical advice and standards. More examples from the table can be found in Table 8.

**Table 7.** Example of a message (relating to diet) from the table of content.

Area	TDF	BCT	Barriers	Enablers	Content	Audience	Format
Diet	Social influences	Modelling behaviour	Hospitality: being expected to eat sweets/rich food at social occasions such as weddings		Scenario: A person newly diagnosed with diabetes goes to a wedding and tries to resist large amounts of *biryani* and sweets. Friends pressure him to eat them.	Men and women Diabetic	Drama
					Strategies used by the person with diabetes: - Requests a smaller portion and does not have any sweets - Explains to his friends why he needs to be careful and control his diabetes		
					Other messages: - Everyone needs to be careful about what they eat - It is important to be supportive of people and allow them to have smaller portions/have alternatives to rich and sweet food

**Table 8.** Further examples of messages from the table of content for script writers.

Message no	Focus area	Aspect	Communication objective (BCT)	Barrier	Enabler/motivator	Content/key message		Audience	Format	Comments
48	General	Memory, attention and decision making Environment	Modelling behaviour	Difficulties in maintaining a routine Poverty makes it difficult to maintain a moderate, regular lifestyle	Routine, balance and moderation = healthy lifestyle Lifestyle changes are not too complicated and within peoples’ reach	Scenario: conversation between someone who is drinking *lal* cha with no sugar and a friend/relative about healthy lifestyles.		Men and women Non-diabetic	Drama/conversation	Could have a woman and/or an older person
						Friend	Response			
						Why are you drinking lal cha and no sugar?	I try not to have too much sugar. I also try to eat regularly and not too much. If I do have sweets just have a little bit. I don’t have oily food at home, but on certain occasions when I have *pilou* I will just have a little bit.			
						So you try to keep to a routine?	When I can. I walk in the morning, eat regularly and have a balance of different types of food. I don’t smoke or eat many snacks.			
						Is it hard to have this lifestyle?	No, you just have to be careful. When you start it is possible to continue. It is good for all the family, we all eat a moderate, balanced diet and I walk with my sister.			
						When you are working can you do this?	Yes. I just plan things around my work. Everyone rich and poor needs to think about how they can have a healthy lifestyle and plan accordingly.			
						And do you keep healthy?	Yes the doctor said I am very well, and I feel healthy. My blood pressure is good and I don’t have diabetes.			
						Oh I have high sugar, or pre-diabetes, can I still do this?	Yes of course! See your doctor, but eating well and exercising regularly is important way of managing your health			
						Key messages: - Strive for a moderate lifestyle; regular exercise and eating reasonable amounts, having small amounts of ‘unhealthy’ food - lifestyle changes are within people’s reach: rich and poor, male and female. - They help keep you healthy and manage illness				
68	General (Diet, smoking)	Beliefs about consequences Environment, context and resources Social influences	Shaping knowledge	Religious beliefs and fate Poverty makes it difficult to maintain a moderate, regular lifestyle	Religious beliefs and responsibility Routine, balance and moderation = healthy lifestyle Lifestyle changes are not too complicated and within peoples’ reach Balance in food considered to be good Religion discouraging smoking	Doctor and an imam: - A balanced, regular lifestyle is healthy for diabetics and non-diabetics - Try to eat a balanced diet in moderation - Exercise everyday - Try to avoid bad habits like smoking and smokeless tobacco products - Draw on support from your families and communities - These changes are possible for everyone, rich or poor - Islam teaches we need to look after our bodies and health - We can change ourselves, our families and communities		Men and women Diabetic and non-diabetic	Straight information	Motivational message to encourage a moderate, healthy lifestyle
49	Care seeking	Beliefs about consequences	Health consequences Shaping knowledge: complications of diabetes	Wait until diabetes is bad before seeking care		The story of a diabetic: I suffered from some of the complications of diabetes. I went to the doctor due to having problems with my feet being red, warm, swollen and with cracks. The doctor helped treat my feet but also sent me for a blood test. I found I had diabetes. The doctor helped me to understand I can control my diabetes with medicine, diet and later exercise. If diabetes is left untreated like mine you can get many complications. Such as: foot problems, problems with eyesight, nerve damage and kidney damage. These are all very serious. The good news is that diabetes can be managed and prevented. It is better to seek help and make lifestyle changes before you get very sick Key points: If you delay seeking care and have diabetes you can get complications The complications of diabetes Diabetes can be prevented and managed		Men and women Diabetic	Personal story/account	A personal story or account about diabetes might motivate people to seek care and try to prevent diabetes It would be good if at least one of message 49 and 50 could be a female
72	Care seeking	Behavioural regulation	Goal setting		If treatment is planned in stages patients more likely to return and not feel overwhelmed	If you have diabetes it is sometimes easier to plan your treatment/behaviour change in stages. Talk to your doctor about this. - Example of planned behaviour could be: - Planned behaviour: - At the beginning: - Take medicine as advised - Exercise when can - Slowly reduce portions, fat and sugar - After a couple of weeks: - Walk everyday - Weigh self regularly - Talk to family about diet and cooking - Take medication - After one month: - Walking half an hour everyday - Family changing eating habits - Reduced fat, sugar and portion sizes - Taking medication regularly - Continue going for testing		Diabetic Men and women	Pros and cons	Check with medics if this is feasible We could also say that anyone can plan to change their behaviour
51	Exercise	Professional role and identity Emotion, Skills	Shaping knowledge Health consequences	Lack of knowledge that exercise can help prevent diabetes Able to integrate walking into everyday routine People who exercise seen as educated Not walking seen as ‘lazy’ Too cold to swim	Lack of time to exercise/walk People who exercise seen as educated Not walking seen as ‘lazy’ Men: Swim, do push-ups, walk, some sports in the past Women: walk, stretch, swim	Exercise is very important. Exercise will make your heart beat faster and increase blood flow and oxygen to your muscles and organs. It can help to prevent diabetes, control your weight, as well as decrease the risk of heart problems and blood pressure and is good for your general well being. It also helps to control diabetes. Exercise stimulates brain chemicals and can make you feel happier. Exercise is for not just for the educated and affluent. It should be done whether you have diabetes or not, are rich or poor, old or young, man or woman. Everyone should aim to do half an hour exercise a day. It is important to make exercise a priority and try to do some everyday. Everyone can find a type of exercise they like or can do. There are many types of exercise. Exercises include: walking, running, sports, swimming, riding bicycle etc. If it is too cold to swim, walk. You can try different types of exercises too. Make time to exercise – try to walk places instead of getting transport, instead of watching TV do some exercise!		Men and women Diabetic and non-diabetic	Dr: Straight information	
52	Exercise	Environment, Social influences, Memory/attention, Emotions	Modelling behaviour Social support	Women feel judged/shamed walking outside Women feel unsafe walking alone Lack of time to exercise/walk Exercise viewed as not a normal thing to do	Walking with other people Walking with friends feels good Able to integrate walking into everyday routine	Scenario Two females walking together – they talk to another female neighbour and try to encourage her to join them.		Women Non-diabetic	Drama	Keep as females: it would be good if we can try and encourage females to encourage each other
						Question	Response			
						Where are you walking?	We are walking for health reasons. We also enjoy walking together.			
						Do you have diabetes?	No. It is always good to walk. Walking can prevent diabetes.			
						As a woman doesn’t it look bad? Do you feel safe?	Women need to walk too. By walking together we feel safer. If women all walk then we will change how people think about women walking. We see exercise as a normal thing to do.			
						Do you have time to walk?	We walk every morning as part of our routine. We also think of when we can walk – sometimes we walk instead of getting transport.			
						Is it not tiring?	At first it was hard, but now we enjoy it and you feel better in the long-term. It is also fun to walk together			
						Final message – try to encourage other women to walk, that way everyone will benefit and we can change public responses. Exercise will then be seen as normal.				
57	Stress	Environment, context Memory, attention and decision making	Pros and cons	Not dealing with the root causes of stress Coping mechanisms: smoking, taking pills		In the last message we spoke about ways people deal with stress. Now we are going to talk about the pros and cons (good and bad points) about each. Some people when they are stressed will take sleeping pills, smoke and take pain killers. Pros/why you do it: The reasons people do this are: - will help them sleep sometimes and take away pain - gives some relief in the short term Cons/why you maybe shouldn’t rely on this: - These might help in the short term but won’t help in the long-term - Smoking is very bad for your health - Taking too many pills can be harmful (Pros outweigh pros) The other coping mechanisms: talking to others, exercising, looking at the bigger picture, exercise and some religious rituals. Cons/reasons people don’t it: - Might be harder to do in the short term - May not think of it/may not be obvious Pros/reasons to do them: - Better long-term solutions that you can do - Exercising is good for your physical health - Talking to people you trust can help relations and give long-term support (Pros outweigh cons)		Men and women Diabetic and non-diabetic	Straight information	Could be a doctor
58	Stress	Social influences	Modelling behaviour Social support	Others will know their problems if they talk about them	Coping mechanisms: talking to others People of the village come together to help those in need e.g. if sick	Scenario: female very distressed because her son is sick and she has money problems. She has a discussion with her neighbours who are very supportive and offer support, encouraging her to talk to them and they offer practical support. - Key messages: - support one another - sometimes just listening helps - talk to people you trust		Men and women Diabetic and non-diabetic	Drama	
60	Diet	Environmental context and resources Memory, attention and decision Social influences Beliefs about consequences	Modelling behaviour Pros and cons	Availability of ‘unhealthy’ food inside and outside the home Convenience of eating outside the home Habit of snacking inside and outside the home Social gatherings and meeting in tea shops	‘Home-cooked’ food believed to be healthy and ‘outside’ food unhealthy	Scenario: two men outside of the home and one wants to go for snacks and sweet tea, the other wants to go home and have food at home. They discuss the pros and cons of eating outside the home and		Men Non-diabetic	Drama	
						1^st^ man: pros of eating outside	2^nd^ man: response, cons			
						There is so much food outside, it is very convenient	Yes that is true, but a lot of it is also fried and unhealthy. At home we can decide better what to eat.			
						The fried food such as *shingaras, puris* etc. are so tasty	Yes that is true. But too many are unhealthy. We can try to only go there occasionally.			
						But I am in the habit of going to the tea shop and it is very sociable, we see are friends there.	Ok, lets go and just get some *lal cha* without sugar. We can still see our friends. Later we can eat at home.			
						But at home there is also unhealthy food!	This is true! But we can decide better what we eat. Let’s go to my house and we can have some fruit and *lal cha* and then later we can have some dinner.			
						Key messages: - moderate the amount of time you eat outside the home - you can go out and eat less fried food - at home you have more control over what you eat				
61	Diet	Social influences	Shaping knowledge Social support	Cooking: women may cook with lower levels of oil etc. Family not supporting different/healthy eating habits	Cooking: women cooking with high levels of oil etc., men asking for it Family supporting different/healthy eating habits Shopping: men shop, women can intervene	Straight information: Families usually eat together. Everyone in the family has a role in eating well and changing eating habits. They include:		Men and women Non-diabetic and diabetic	Straight information	Could be a song, poem, or different voices from a family or a doctor
						Men	Men/husbands usually shop. Talk to your wife about what you will buy. Try to buy lots of vegetables and fruit. Get vegetable oil instead of *ghee*. Get cheaper more healthy sources of protein such as fish, eggs and chicken instead of meat. If possible get brown rice and flour.			
						Women	Women/wives/daughter in-laws do most of the cooking. Talk to your husband about what they should buy. Try to cook with lots of vegetables and make salads. Only cook with as much oil as you need. Avoid deep frying food. Explain to your family why you are cooking like this. Food can still be tasty with lots of flavour.			
						Older: in-laws	You have lots of influence. Encourage members of the family to shop and cook well. Praise healthy food and cooking.			
						Younger: children	You can encourage your parents to shop and cook well. Explain to them why it is important.			
						Key messages:All the family need to be involved in changing eating practices - Good cooking and eating practices benefits the whole family				
74	Smoking	Knowledge Social influences	Shaping knowledge Health consequences	Overall lack of awareness regarding the link between smoking and ill health. No awareness of the link between smoking and diabetes Smoking perceived to be common among certain groups: farmers, younger people etc.	Stigma: smoking in front of elders, women etc. Not acceptable to smoke in public spaces (bus, mosques etc) Family: less likely to smoke if it is not done in the family Family: discouraging smoking/encouraging to give up Peer pressure to smoke Religion discouraging smoking Quitting because of work Less likely to smoke with age	Straight information about smoking and perceptions of smokers: - Perceptions of people who smoke: mostly men, young, farmers, students, rural areas. - It is more men than women who smoke, but all different types of people smoke. They also may take other tobacco products. - There is some stigma regarding smoking, for example people will not smoke in front of their elders, women and in spaces such as the mosque. - It is important not to smoke in front of people as passive smoking can also cause harm to those around them. - Family and peer groups have an important role in not smoking – people are less likely to start smoking if their friends and family don’t smoke. We can also encourage each other to stop smoking. - People may also give up smoking because of their age, health, religious reasons and because of their work. - It is good to be motivated to give up smoking. There are also health consequences of smoking. - Reminder of the consequences of smoking: - People who smoke are at increased risk of diabetes, cancer, lung diseases, heart diseases, brain stroke and poor circulation, erectile dysfunction in males and infertility in women, tooth and gum disease. - Diabetics who smoke are less able to control their diabetes. Smokers with diabetes have a higher risk for complications of diabetes such as: heart and kidney disease, poor blood flow in the legs and feet that can lead to ulcers and infections, eye problems and damaged nerves		Men and women Diabetic and non-diabetic	Doctors voice	Perceptions of smoking and stigma is taken from the formative research
77	Smoking	Beliefs about capabilities Memory, attention and decision process Social influences	Modelling behaviour Shaping knowledge Social support	Belief that can only give up smoking by quitting completely Addiction to smoking	Family: less likely to smoke if it is not done in the family Family: discouraging smoking/encouraging to give up	Scenario: an uncle discovers his nephew smokes. His nephew admits he does but does not know how to stop as he is addicted. The uncle offers family support and also gives tips for giving up smoking. - Tips for giving up smoking: - You need to be personally motivated, think of the reasons you don’t want to smoke - Distract yourself with other things: talk to other, sport, work - You can cut down slowly the number of cigarettes you smoke every day until or stop completely - Keep trying eventually you will be able to give up - You can try and stop smoking with someone else - Change routines associated with smoking/do other things: exercise, *namaj*, talk to people - Spend time with friends/family who do not smoke - Key messages: - ways to give up smoking - family support to give up smoking		Men Non-diabetic	Drama	

### Message production and delivery

The finalised table of contents was used to guide the intervention; specifically exact information needed to be shared as part of the intervention – and ensured all barriers and enablers emerging from the research were addressed. The table was shared with scriptwriters and a production company who were responsible for the format of the messages and making them both entertaining and understandable. Songs, dramas and straight information were all used with the language colloquial and tailored to the region. Project researchers and clinicians had final editorial control over the messages to ensure they were in-line with the context, and that they represented the content. A total of around 100 unique messages were produced and delivered to approximately 9000 individuals across 32 villages in Faridpur on a twice weekly basis between October 2016 and December 2017.

## Discussion

In response to a lack of guidance in research regarding the development of a theory-driven mHealth intervention rooted in local context, we have developed and applied a six-step process to develop content for mHealth messages related to awareness raising and lifestyle changes for prevention and control of diabetes in rural Bangladesh. The process involved integrating in-depth qualitative contextual research with theory. The benefits of the steps outlined in the paper are that they are replicable and hence the model developed can be tested in other contexts. The exact methods used in the formative research do not need to be replicated, however contextual research identifying enablers and barriers to behaviour change is important. TDF and the corresponding BCTs can be applied to identify barriers and enablers to behaviour change. Hence the steps provide a guideline to intervention development, and due to the comprehensive nature of TDF and BCTs there is room for flexibility regarding the problems the interventions may address and the techniques that can be implemented to address them. The effectiveness of the messages is yet to be tested through the outcome of the trial and if applied in other contexts.

Many behaviour change interventions are targeted at individuals, and in those cases a clear target and pathway of change may be needed. For example, according to the transtheoretical change model, change is assumed to follow certain stages through which they are targeted [5,13]. While other models may account for wider societal and higher level influences (social ecological model for example) [25], pathways, influences and beliefs vary widely not just according to individuals, but also groups. Behaviour and behaviour change is complex, having multiple targets is more complex: based on our experiences we believe it would be difficult for a classic, single theory to address these challenges. While the broadness of TDF, COM-B and BCTs have been criticised for not being specific enough, we found this to be a strength when applied to a population level intervention as it means a range of strategies and processes could be applied – increasing the likelihood of appealing to different segments of the population. For example we could classify problems specific to both genders and find appropriate BCTs to address them. Furthermore, within the range of the domains and BCTs, specific needs and approaches could be addressed according to specific barriers and enablers. We found TDF and the process we developed useful in enabling us to break down the specific needs of a population, identify what needs to change and what can be built upon and identify techniques in which this could be achieved.

An important aspect of the intervention was the packaging of the messages – with a production company being responsible for this. It was therefore important to convey the primary research effectively so that it could be applied appropriately. We did have a discussion about the scriptwriters conducting the qualitative research, in order for them to have a detailed understanding of the context. However, this would have meant them needing to be trained in qualitative research methods and be willing and able to spend time in the field. In practice members of the research team were more involved than planned in the editing and production of the messages – in order to ensure context was appropriately conveyed. Lessons learned from this collaboration were: collaborations and communication need to be carefully thought through and given plenty of time, as well as considering very early on in the process what collaborators of different background need and expect from each other and consider creative ways of achieving this (for example scriptwriters spending time in the field, and researchers learning how to write scripts).

### Limitations

There were limitations to the study and the intervention. The broadness of the TDF and COM-B frameworks makes it difficult to unpick and assess exactly what aspects of the theory were effective. However, for the purpose of message development at a population level having a broad theoretical framework was useful (as explained in the discussion) and therefore for this study the benefits of the broadness of the models outweighed their potential weakness. Furthermore, as part of the trial we did conduct a process evaluation, which may illuminate what aspects of the intervention worked well and what did not. We were also limited by the nature of the mHealth intervention, as we were very limited in the behaviour change techniques that could be applied, and the intervention lacked two-way interaction.

## Conclusion

A replicable process for developing the content of voice messages (and perhaps other interventions) for behavioural change, grounded in both theory and in-depth research, has been developed. Through identifying specific barriers and enablers to behaviour change from contextual research and categorising them according to the transtheoretical domain framework, BCTs can be applied to the barriers and enablers to promote behaviour change. While the process requires thorough research, clear outcomes and an application of TDF, the packaging of the intervention is also important. The six-step process developed is also significant as it is, to the best of our knowledge, the first to apply TDF and the COM-B model in a low-income setting. Thus it is particularly important that the local context is considered, and the behaviour change approaches contextualised appropriately. Ultimately the results of the trial and on-going evaluation will indicate the effectiveness of the intervention and its development, but the deep understanding of the intervention and the design decisions underpinning it will contribute enormously to the interpretation of the trial findings.

## Paper context

The evidence for the effectiveness of mHealth interventions in low-income countries is somewhat limited, and many lack a theoretical basis and context is not always considered. This paper addresses some of the gaps in research – it reports the process of applying qualitative research to behavioural theory to guide the development of an mHealth intervention in Bangladesh. It is hoped that the principles and process developed will be applied and tested in other contexts.
